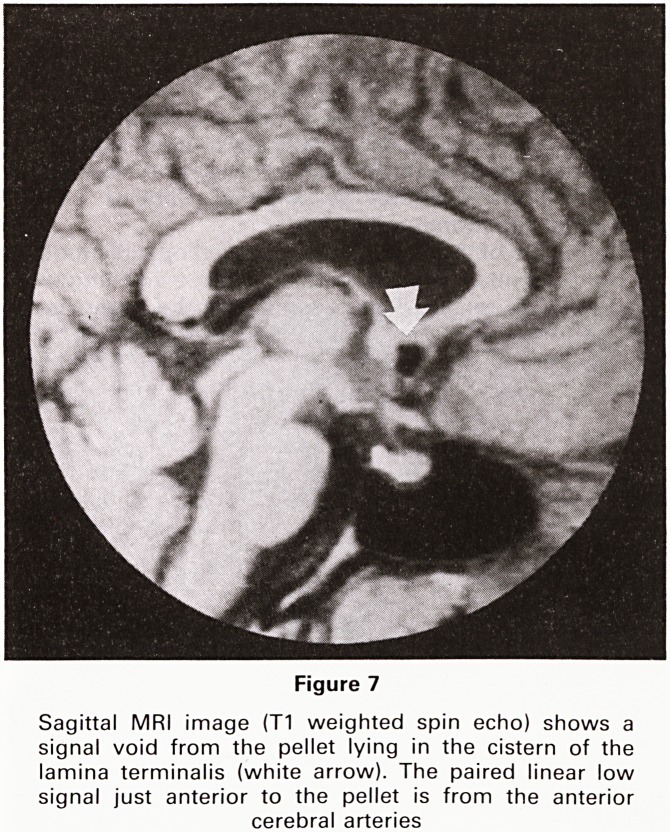# Magnetic Resonance Imaging of the Orbit

**Published:** 1988-05

**Authors:** A. Berger, T. Lewis, P. Goddard


### Magnetic Resonance Imaging of the Orbit

**A. Berger BSc**

**P. Goddard MD FRCR**

**T. Lewis FRCR**

### INTRODUCTION

The initial impact of Magnetic Resonance Imaging (MRI) was in neuroradiology, where its detection of pathology and demonstration of normal anatomy presented clear advantages over the other available techniques. Its use in ophthalmology is more recent and its advantages over Computed Tomography (CT) were initially limited by the relatively low resolution of early machines. With improvements in hardware and software it has become increasingly obvious that MRI has much to offer in the investigation of orbital pathology. We wish to demonstrate, using two cases, the benefits available from orbital MRI.

**Case Histories**

1. David, aged 7, presented with a 4 week history of right proptosis. His proptosis progressed over the following week and he became unwell with headaches, fever, vomiting and noisy respiration. Examination under anaesthetic revealed a white friable tumour within the ethmoid sinuses. Histology showed this to be an embryonal rhabdomyosarcoma.

Plain x-rays of the facial bones showed opacification of the right maxillary antrum with soft tissue swelling over the orbit (Figure 1). CT suggested that the mass arose from the medial rectus muscle, involving ethmoid and sphenoid sinuses and extending back to the orbital apex (Figure 2). The possibility of intracranial extension was debated.

An MRI scan was performed and this showed the tumour with considerable clarity (Figures 3 and 4). The lesion was shown involving the paranasal sinuses and the medial part of the right orbit. The right medial rectus muscle could not be separated from it. Retro-orbital extension of tumour was not seen.

The MR images were used as an adjunct to the CT in planning radiotherapy. Additionally David has received intravenous and intrathecal chemotherapy.

**Figure 1**

(Case 1) Frontal projection of the paranasal sinuses showing an opaque right antrum and increased radiodensity in the right orbit due to soft tissue swelling

**Figure 2**

Axial CT scan (GE9800, 3 mm slice thickness) shows right proptosis with a soft tissue mass destroying the lamina papyracea and involving the medial part of the orbit and right ethmoid air cells

**Figure 3**

Coronal T1 weighted spin echo MR image demonstrating antral and ethmoid invasion and involvement of the medial rectus muscle

**Figure 4**

A sagittal image using the same sequence shows the posterior extent of the tumour

2. Mr L. aged 19, was accidentally shot through the left eye with an air gun pellet. When seen in the Bristol Eye Hospital casualty department he was alert and orientated, though complaining of headache and epistaxis and blind in his left eye. Clinical examination showed a laceration of the lower lid with rupture of the globe, vitreous prolapse and hyphaema of the eye.

Plain skull x-rays showed the air gun pellet within the vault above the sella with a small fragment close to the orbital apex (Figure 5). A CT scan also showed the suprasellar location of the pellet but could not provide accurate enough localisation for surgical planning (Figure 6). MRI was requested for better pre-operative evaluation.

Metallic objects are usually considered an absolute contra-indication to MRI scanning and we devised several tests to assess the safety of the procedure before the scan. These are the subject of a separate case report to be published elsewhere.

The scan demonstrated the foreign body (shown as a signal void) lying within the cistern of the lamina terminalis immediately posterior to the anterior cerebral arteries (Figure 7). Other images showed high signal from the inferior part of the left frontal lobe indicating focal contusion.

**Figure 5**

(Case 2) A lateral skull x-ray shows the radio-opaque air gun pellet (white) above the sella, with a second, smaller, fragment near the orbital apex

**Figure 6**

Axial CT (Siemens DRH, 8 mm slice thickness) shows the foreign body but little further anatomical detail

**Figure 7**

Sagittal MRI image (T1 weighted spin echo) shows a signal void from the pellet lying in the cistern of the lamina terminalis (white arrow). The paired linear low signal just anterior to the pellet is from the anterior cerebral arteries

In view of the risks involved in its removal from a relatively inaccessible site the pellet has been left within the skull. No vision has returned to his left eye and future enucleation is planned.

**Discussion**

The orbit, in particular, is well suited to MRI where the advantages of multiplanar imaging are particularly apparent. MRI provides good visualisation of the optic nerve and bulbar muscles and is the only available modality for imaging the intracanalicular parts of the optic nerves. Good images of the parasellar region and chiasm are also routinely obtained and provide further valuable information. Thin sections (3–5 mm) in the axial and coronal planes are routinely used in conjunction with a closely applied receiver coil (surface coil) which allows greater spatial resolution than the standard head coil used for intracranial imaging. Sagittal images (as used in both of the above cases) may be invaluable.

Cortical bone is not shown on any MR sequence and bone is only visualised by its marrow and fat content or as a signal void. Thus in Figure 3 the orbital roofs are shown as a black line between the orbital fat (white) and inferior parts of the frontal lobes (grey). The lamina papyracea is bounded by fat on its lateral border and air medially and therefore cannot be identified. The same is true for the orbital floor. The intra-orbital structures (globe, nerves, muscles and blood vessels) are seen as low signal structures surrounded by high signal fat. A kind of "spatial blurring" due to the different signal frequency of fat from other tissues ("chemical shift") produces some image degradation but a fat supression sequence (STIR) is available which reduces this problem.

The increased resolution using surface coils allows other structures to be demonstrated: the sclera, choroid/retina, iris, lens, ciliary body, vitreous, lacrimal gland and blood vessels can be identified.

Such imaging, provides anatomical detail often superior to that achieved by current CT scanners though at the expense of a slightly longer scanning time. The absence of harmful side effects at the field strengths used for imaging is a considerable advantage over conventional thin section axial CT, where cataractogenic radiation doses can occur with repeat examinations.

**Conclusion**

Magnetic Resonance Imaging has considerable potential in the investigation of orbital pathology. MRI has been shown to be useful in demonstrating a variety of orbital tumours, infections, scleritis and in trauma because of the high contrast, resolution and multiplanar imaging capability.

**Acknowledgements**

We would like to thank Drs Mott and Duncan from the Bristol Children's Hospital and the Ophthalmologists and Neurosurgeons involved in the care of these patients for referring them for investigation with MRI. We would also like to thank the staff of the Bristol MRI centre, where the scans were performed.

## Figures and Tables

**Figure 1 f1:**
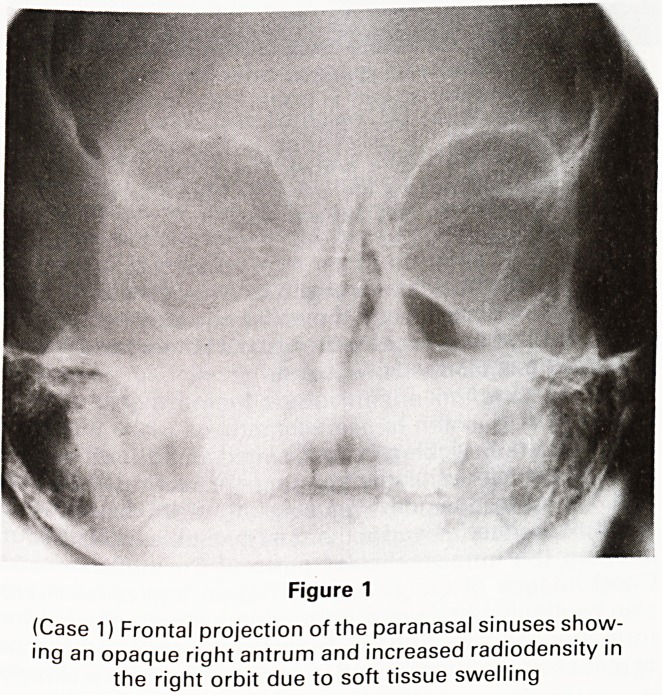


**Figure 2 f2:**
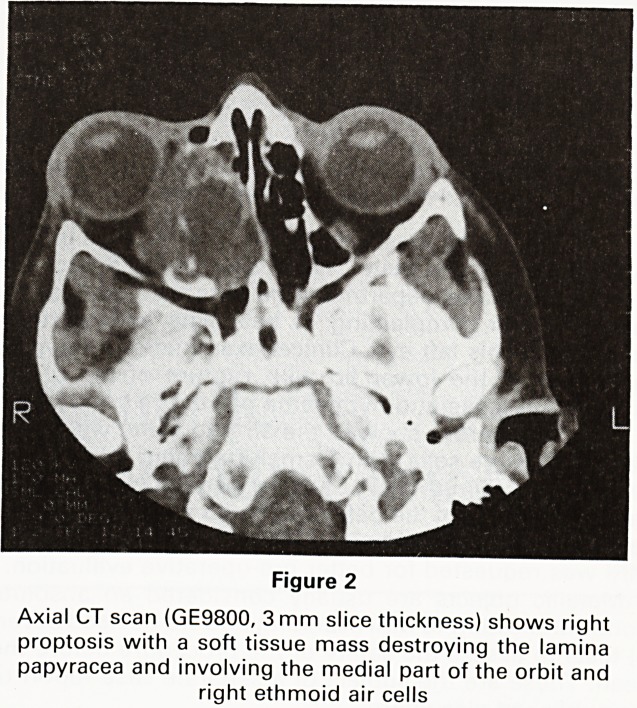


**Figure 3 f3:**
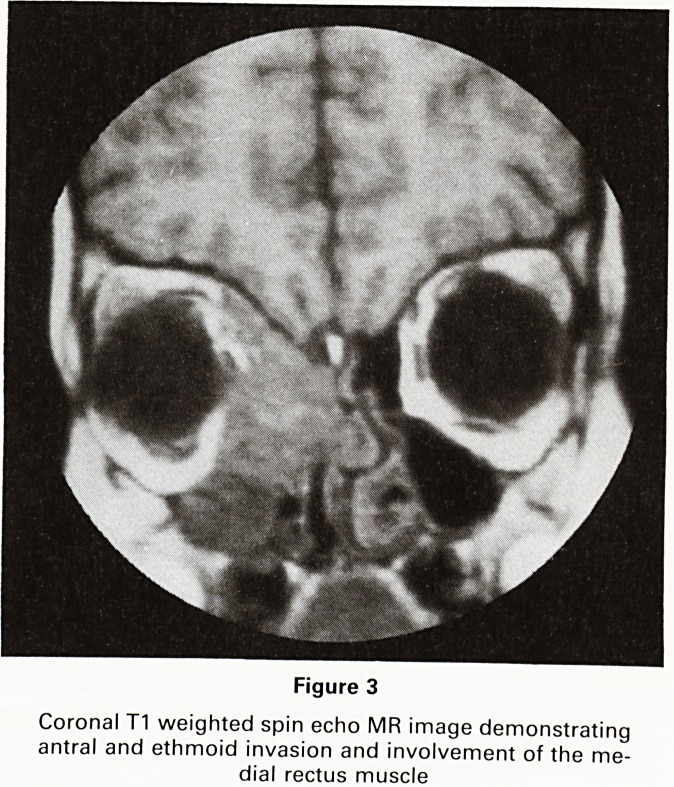


**Figure 4 f4:**
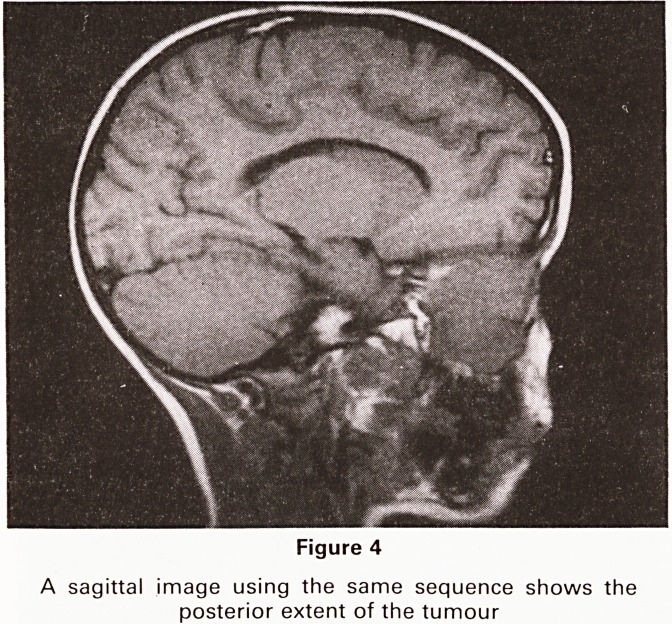


**Figure 5 f5:**
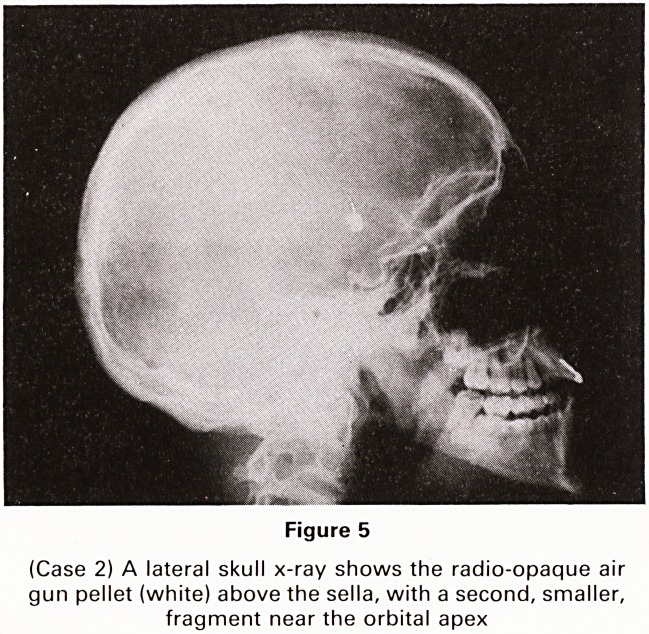


**Figure 6 f6:**
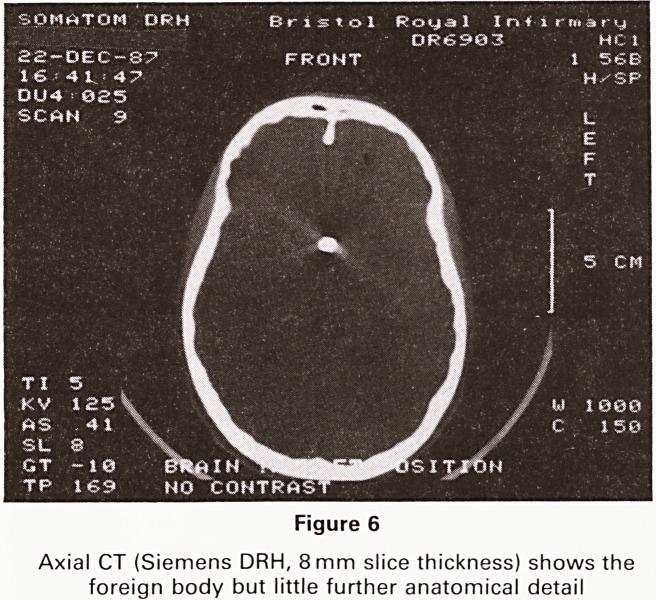


**Figure 7 f7:**